# METTL5 in physiology and pathology: mechanisms and implications

**DOI:** 10.3389/fcell.2025.1708541

**Published:** 2025-12-18

**Authors:** Chunhong Li, Xiulin Jiang, Yixiao Yuan, Qiang Wang

**Affiliations:** 1 Department of Oncology, Suining Central Hospital, Suining, Sichuan, China; 2 Department of Systems Biology, City of Hope Comprehensive Cancer Center Biomedical Research Center, Monrovia, CA, United States; 3 Department of Gastrointestinal Surgical Unit, Suining Central Hospital, Suining, Sichuan, China

**Keywords:** cancer progression, immune microenvironment, METTL5, RNA methylation, rRNA modification, therapeutic target, tumor immunity

## Abstract

METTL5, in complex with TRMT112, catalyzes N^6^-methyladenosine (m^6^A) at A1832 of 18S rRNA, acting as a novel regulator of translational control. This modification within the ribosomal decoding center modulates ribosome assembly and selective mRNA translation. Physiologically, METTL5 is required for embryonic and neural development, stem cell fate, spermatogenesis, and cardiac function. Aberrant expression has been reported in multiple cancers, where it correlates with poor prognosis. Mechanistically, METTL5 drives proliferation, metastasis, and chemoresistance by promoting oncogenic translation, reprogramming metabolism, regulating ferroptosis, and shaping the immune microenvironment. Cooperation with m^6^A readers, including IGF2BP and YTHDF proteins, contributes to these effects. Targeting METTL5 shows therapeutic promise, with compounds such as salvianolic acid C and scutellarin demonstrating inhibitory activity. In this review, we summarize the molecular characteristics, physiological roles, and pathological functions of METTL5, highlight its mechanisms in tumorigenesis and immunity, and discuss its potential as a biomarker and therapeutic target.

## 1 Introduction

RNA modifications constitute an essential layer of post-transcriptional gene regulation and have emerged as critical determinants of RNA structure, stability, and function (Barbieri and Kouzarides, 2020). While N6-methyladenosine (m^6^A) on mRNA has been extensively studied, chemical modifications on non-coding RNAs-particularly ribosomal RNA (rRNA)-are equally crucial for cellular homeostasis (An and Duan, 2022). rRNA modifications contribute to ribosome assembly, fine-tuning of ribosome architecture, and modulation of translational initiation and elongation (Milenkovic and Novoa, 2025). Advances in high-throughput sequencing, mass spectrometry, and structural biology have revealed that distinct rRNA modification patterns can give rise to “specialized ribosomes,” which selectively regulate the translation of specific subsets of mRNAs in development, stress responses, and disease processes.

METTL5 was recently identified as the methyltransferase responsible for catalyzing m^6^A modification at a specific site on human 18S rRNA, with its enzymatic activity dependent on the cofactor TRMT112 (Dai et al., 2023). Functional studies have shown that METTL5-mediated rRNA methylation alters the local structural environment of the small ribosomal subunit and impacts the translation of particular mRNAs, potentially through modulation of initiation complex assembly or recognition of specific 5′UTR features (Turkalj and Vissers, 2022). *In vivo* models further support an essential role for METTL5 in development-especially neurodevelopment-where METTL5 loss leads to growth defects, impaired cognitive function, and altered stress responses (Zhang et al., 2025). Emerging evidence also indicates that aberrant METTL5 expression or activity may contribute to tumorigenesis by influencing cancer cell proliferation, metabolic rewiring, and therapeutic sensitivity, highlighting its potential as both a biological regulator and a disease-associated factor (Tooley et al., 2023).

Despite these advances, several key questions remain unresolved. The mechanistic basis by which METTL5-dependent rRNA m^6^A selectively regulates translation of defined mRNA subsets remains incompletely understood, and whether this involves structural remodeling of the ribosome or altered interactions with translation factors is still under debate. The extent to which METTL5 contributes to ribosome heterogeneity across tissues, developmental stages, or pathological conditions also remains unclear, as does the conservation of these effects across species. Moreover, whether METTL5 possesses additional RNA substrates or non-catalytic regulatory functions has not been fully explored. Translating current mechanistic insights into clinical applications-for example, leveraging METTL5 as a biomarker or therapeutic target-also faces substantial conceptual and technical challenges.

Given these knowledge gaps, this review aims to provide a comprehensive synthesis of recent advances in understanding METTL5-mediated rRNA methylation and its physiological and pathological implications. We first discuss the structural and biochemical characteristics of METTL5 and its interaction with TRMT112, followed by an overview of its roles in ribosome function and translational regulation. We then evaluate current evidence linking METTL5 to human diseases, with a particular focus on neurodevelopmental disorders and cancer. Finally, we highlight outstanding questions, technological limitations, and promising future directions for the field. Through this structure, we aim to provide a coherent framework that clarifies the motivation and significance of studying METTL5 and outlines the most compelling avenues for future research.

## 2 Molecular characteristics and biological functions of METTL5

### 2.1 Gene localization and structural features

The human METTL5 gene is located on chromosome 2q31.1 and encodes a protein of approximately 209 amino acids (van Tran et al., 2019). METTL5 belongs to the S-adenosylmethionine (SAM)-dependent methyltransferase superfamily and contains a typical Rossmann-like fold that enables binding to SAM as the methyl donor. Similar to many other methyltransferases, the catalytic activity of METTL5 strictly depends on the formation of a stable heterodimer with the small protein TRMT112 (van Tran et al., 2019). TRMT112 plays a crucial role in maintaining the structural stability of METTL5 and facilitating its enzymatic activity. Crystallographic studies have revealed that the METTL5–TRMT112 complex harbors a conserved SAM-binding pocket and catalytic core, ideally suited for the site-specific recognition of rRNA substrates (van Tran et al., 2019). Recent studies confirmed that the METTL5–TRMT112 complex deposits an m^6^A modification at position A1832 of human 18S rRNA. TRMT112 is indispensable for the stability of METTL5, and mutations in METTL5 associated with microcephaly and intellectual disability disrupt this protein–protein interaction, underscoring its pathological significance in neurodevelopment (Sepich-Poore et al., 2022). Functionally, the loss of METTL5 leads to translational reprogramming in both cancer cells and mouse models, with Mettl5-deficient mice displaying reduced body size and metabolic defects (Sepich-Poore et al., 2022).

### 2.2 Function as an 18S rRNA m^6^A methyltransferase

Independent studies have consistently established that 18S rRNA is the sole substrate of METTL5. Specifically, METTL5 catalyzes N^6^-methyladenosine modification at position A1832 of the small ribosomal subunit, located near the h44 decoding center. This modification is highly conserved across species, suggesting its fundamental importance in ribosome function (Sepich-Poore et al., 2022). Both *in vivo* and *in vitro* experiments have demonstrated that loss of METTL5 or its partner TRMT112 leads to a complete or dramatic loss of m^6^A modification at A1832, confirming that the METTL5–TRMT112 complex is the exclusive catalytic machinery. Given that this site resides in the decoding center, m^6^A modification is thought to modulate local ribosomal conformation and interactions, thereby fine-tuning ribosome–mRNA recognition and translational fidelity (Sepich-Poore et al., 2022).

### 2.3 Regulation of translational efficiency and protein synthesis

The A1832 site lies adjacent to the ribosomal decoding center, a critical region for codon–anticodon pairing between mRNA and tRNA. Consequently, METTL5-mediated m^6^A modification can influence ribosome conformation and modulate both translation initiation and elongation (Rong et al., 2020). Interestingly, although global translation is not completely impaired in METTL5-deficient cells, the translational efficiency of a subset of transcripts is markedly reduced. These transcripts are often enriched for genes related to cell proliferation, differentiation, stress responses, and cancer-associated pathways (Rong et al., 2020). This indicates that METTL5 primarily regulates “translational selectivity” rather than bulk protein synthesis. For example, loss of METTL5 has been shown to attenuate the translation of oncogenes such as c-Myc, thereby influencing cell growth and tumorigenesis. Furthermore, animal models suggest that METTL5 plays critical roles in maintaining stem cell self-renewal, developmental processes, and neural system function (Xia et al., 2023).

### 2.4 Distinction and interplay with other methyltransferases

Compared with canonical m^6^A writers such as METTL3/METTL14, METTL5 exhibits several notable differences. First, substrate specificity diverges completely: METTL5 exclusively modifies 18S rRNA, whereas METTL3/METTL14 predominantly act on internal mRNA sites (Sepich-Poore et al., 2022). Second, the complex composition differs: METTL5 requires TRMT112 for stability and activity, while METTL3/METTL14 depend on cofactors such as WTAP, VIRMA, and RBM15 to form a larger complex (Sepich-Poore et al., 2022). Third, the mechanisms of substrate recognition are fundamentally distinct: METTL5 directly targets the ribosomal decoding center to modify the translational machinery itself, whereas METTL3/METTL14 regulate gene expression by altering mRNA stability, processing, and translational efficiency (van Tran et al., 2019). Despite these differences, the two systems are not functionally isolated. Rather, they may act in complementary and synergistic manners: modifications at both the mRNA and ribosomal levels collaboratively shape the translational landscape, thereby exerting profound effects on cell fate decisions and disease progression.

## 3 Physiological processes regulated by METTL5

**FIGURE 1:**
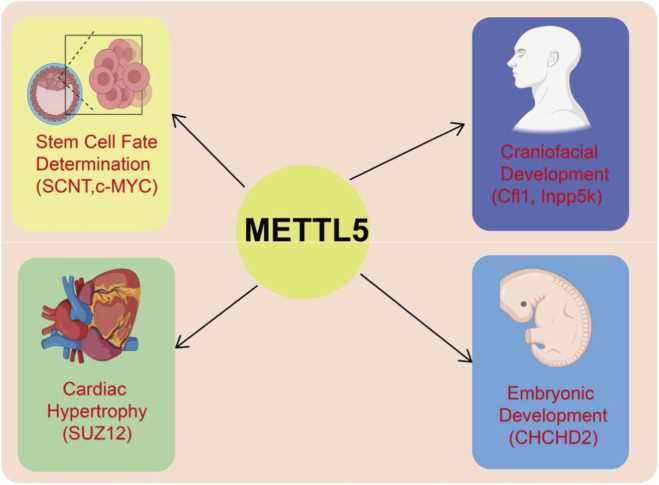
Roles of METTL5 in developmental and non-cancerous processes. METTL5 regulates translation or expression of specific target genes to influence various physiological processes. It contributes to stem cell fate determination (SCNT, c-MYC), craniofacial development (Cfl1, Inpp5k), cardiac hypertrophy (SUZ12), and embryonic development (CHCHD2). Through modulation of these key targets, METTL5 exerts essential roles in cellular differentiation, organogenesis, and tissue development.

As a novel 18S rRNA m^6^A methyltransferase, METTL5 plays essential roles in a wide range of physiological processes. It not only contributes to fundamental biological activities such as craniofacial development, spermatogenesis, stem cell fate determination, and embryonic development, but also regulates fine-tuned functions of the nervous system, including the plasticity of the corticospinal tract. By mediating ribosomal modifications and influencing the translational efficiency of specific genes, METTL5 exerts multilayered regulatory effects across diverse tissues and cell types. Increasing evidence suggests that loss or mutation of METTL5 is closely associated with developmental abnormalities, neurological disorders, and infertility, highlighting its indispensable role in maintaining physiological homeostasis and counteracting pathological challenges. Figure 1 depicts the functions of METTL5 in developmental and non-cancerous processes, highlighting its roles in stem cell fate, organogenesis, and tissue development via regulation of specific target genes.

### 3.1 METTL5 regulates craniofacial development

Craniofacial development is a highly complex and tightly regulated process involving the coordinated proliferation, migration, and differentiation of neural crest and mesodermal cells, ultimately giving rise to the bones, cartilage, muscles, and connective tissues of the head and face (van Gijn et al., 2013). Clinical studies have revealed that mutations in METTL5, the methyltransferase responsible for 18S rRNA m^6^A modification, can result in intellectual disability, microcephaly, and craniofacial anomalies (Lei et al., 2023). Although its precise mechanistic contribution to craniofacial development remains unclear, recent findings show that Mettl5-knockout mice exhibit defective ossification, widened cranial sutures, and phenotypes resembling cleidocranial dysplasia. Further analyses demonstrated that Mettl5 loss increases proliferation but reduces osteogenic differentiation of suture mesenchymal stem cells (SMSCs) (Lei et al., 2023). Mechanistically, the absence of METTL5 significantly suppresses Wnt signaling activity, thereby impairing normal osteogenesis. These results underscore the pivotal role of METTL5 in craniofacial development and SMSC differentiation, and suggest its potential as a diagnostic and therapeutic target in craniofacial malformations.

### 3.2 Spermatogenesis

Spermatogenesis is a highly ordered and intricate process in which spermatogonial stem cells undergo mitosis and meiosis, followed by morphological differentiation to generate mature spermatozoa (Neto et al., 2016). RNA modifications have recently been recognized as critical regulators of gene expression during spermatogenesis. However, the role of rRNA modifications-the most abundant form of cellular RNA modification-remains poorly understood in this context. In a cohort of 1,427 infertile men with oligoasthenoteratozoospermia (OAT), four pathogenic heterozygous variants of METTL5 were identified, all associated with significantly reduced METTL5 expression (Zhang et al., 2025). Functional studies demonstrated that Mettl5-knockout male mice are infertile, exhibiting OAT-like phenotypes accompanied by sperm head and tail abnormalities. While global translation appeared unaffected, the translational efficiency of several spermatogenesis-related genes (e.g., Gk2, Akap4, Fsip2, Odf2, Pgk2) was markedly reduced (Zhang et al., 2025). Clinically, intracytoplasmic sperm injection (ICSI) enabled successful pregnancies in couples where the male partner carried pathogenic METTL5 variants. Collectively, these findings identify METTL5-mediated 18S rRNA m^6^A modification as a novel genetic determinant of OAT and provide new insights into genetic counseling, diagnosis, and potential therapeutic strategies for male infertility (Zhang et al., 2025).

### 3.3 Stem cell fate determination

Stem cell fate determination is a strictly regulated process governed by transcriptional, epigenetic, and translational mechanisms to balance self-renewal and differentiation (Sheikh et al., 2021). Although rRNA accounts for over 80% of total cellular RNA, its modifications and physiological roles remain incompletely characterized. Clinical studies have shown that METTL5 mutations, which impair 18S rRNA m^6^A modification, are associated with intellectual disability, microcephaly, and craniofacial abnormalities (Wang et al., 2022). Protein interaction analyses revealed that METTL5 interacts primarily with RNA-binding and ribosomal proteins. In mouse embryonic stem cells (mESCs), Mettl5 knockout leads to abnormal craniofacial and neural development, and knockout mice display intellectual disability, recapitulating human pathogenic phenotypes. Mechanistically, METTL5 maintains brain function and cognitive development by regulating myelination processes, thereby establishing a causal link between rRNA modification and neural defects (Wang et al., 2022). Recent studies using human induced pluripotent stem cell (iPSC)-derived forebrain organoids further demonstrated that METTL5 deficiency delays neural stem cell proliferation and disrupts the timing of neuronal differentiation, underscoring its role in neurogenesis. Interestingly, translational alterations observed in METTL5-deficient organoids resembled stress responses rather than transcript-specific regulation (Turkalj et al., 2025). Transcriptomic analyses further revealed downregulation of CHCHD2, a mitochondrial gene linked to energy metabolism and neurodevelopment. Overexpression of CHCHD2 rescued the proliferation defects of neural progenitors in METTL5-deficient models. Together, these findings highlight the essential role of METTL5 in coordinating rRNA modification, translation, and metabolism to support stem cell fate determination and human brain development (Turkalj et al., 2025).

### 3.4 Embryonic development

Embryonic development is a complex process in which the zygote undergoes successive cell divisions, migration, differentiation, and organogenesis to form a complete organism (Nehme et al., 2025). As one of the most important epitranscriptomic modifications in eukaryotic RNA, m^6^A plays critical roles in development and disease. METTL5, as the methyltransferase catalyzing m^6^A at 18S rRNA, regulates protein translation and influences embryonic stem cell pluripotency. Notably, Mettl5 expression is significantly upregulated in somatic cell nuclear transfer (SCNT) embryos compared with fertilized controls (Zhang et al., 2022). Based on this observation, researchers hypothesized that Mettl5 knockdown during early SCNT stages might improve developmental efficiency. Indeed, siRNA-mediated suppression of Mettl5 increased blastocyst formation rates and cell numbers (Zhang et al., 2022). Mechanistically, METTL5 inhibition reduced phosphorylation of ribosomal protein S6, decreased repressive histone marks (H3K27me3), increased activating marks (H3K27ac, H3K4me3), and upregulated 2-cell-specific transcripts (Zhang et al., 2022). Additional studies confirmed that Mettl5 deficiency in mESCs results in reduced global translation, spontaneous loss of pluripotency, and impaired differentiation potential. Mettl5-deficient mice exhibited abnormal Mendelian ratios at birth, morphological abnormalities, and behavioral defects resembling human METTL5-associated syndromes, thereby providing a new disease model. Mechanistically, METTL5 facilitates efficient translation of FBXW7, a key regulator of cell differentiation. Loss of METTL5 reduces FBXW7 levels, leading to aberrant accumulation of its substrate c-MYC and delayed initiation of mESC differentiation (Xing et al., 2020). Overall, these studies highlight METTL5 as a pivotal regulator of early embryonic development and pluripotency.

### 3.5 Corticospinal tract

The corticospinal tract (CST) is the principal descending motor pathway of the central nervous system, originating in the cerebral cortex and projecting through the brainstem to the spinal cord to control fine voluntary movements (Welniarz et al., 2017). Following unilateral brain injury, functional recovery of the affected limb critically depends on CST sprouting across the midline into the denervated spinal cord. Although some genes have been identified as regulators of CST sprouting, the role of RNA modifications remains poorly understood (Welniarz et al., 2017). Recent studies investigated whether METTL5 is involved in this process. Overexpression of METTL5 in contralateral cortical motor neurons significantly promoted CST sprouting. Mechanistically, METTL5-mediated 18S rRNA m^6^A modification enhanced the translation of multiple target genes, including Cfl1 and Inpp5k. Increased expression and activation of cofilin, in turn, stimulated actin polymerization and microtubule bundling, thereby facilitating axonal growth (Li et al., 2025). These findings uncover a novel mechanism by which METTL5 regulates axonal sprouting and suggest potential strategies for functional recovery after brain injury.

### 3.6 METTL5 regulates cardiac hypertrophy

Enhanced protein synthesis is a hallmark of cardiac remodeling and hypertrophy. While METTL5 has been implicated in regulating translational efficiency during embryonic differentiation and tumorigenesis, its role in cardiac hypertrophy has only recently been elucidated (Nakamura and Sadoshima, 2018). Using a cardiomyocyte-specific knockout mouse model (METTL5-cKO), researchers found that METTL5 deficiency exacerbated pressure overload-induced hypertrophy and pathological remodeling. Functional assays in primary cardiomyocytes, using both gain- and loss-of-function approaches, further confirmed the regulatory role of METTL5 in cardiac hypertrophy (Han et al., 2022). Mechanistically, METTL5 enhanced the translation of SUZ12, a core component of the PRC2 complex, thereby influencing transcriptional reprogramming during hypertrophic progression (Han et al., 2022). Collectively, these findings identify METTL5 as a novel translational regulator of cardiac hypertrophy via rRNA m^6^A modification, providing new insights into the molecular basis of cardiac remodeling.

Although METTL5 is required for normal development and homeostasis, its mechanism of action in physiological tissues may differ from that in cancer. In normal settings, METTL5-dependent rRNA methylation is thought to support basal protein synthesis and cellular stress responses, ensuring proper organogenesis and tissue maintenance. In contrast, cancer cells frequently exploit METTL5 to sustain oncogenic translation programs, metabolic adaptation, and immune evasion. For example, the METTL5-ATF4 axis becomes selectively amplified in tumors to suppress ferroptosis and resist T cell–mediated killing, whereas this pathway may be less dominant in normal cells. Moreover, cancer-specific regulatory networks, such as aberrant transcriptional activation or altered metabolic states, may enhance METTL5 dependency. Therefore, while the core enzymatic function may be shared, the downstream targets, regulatory context, and biological consequences of METTL5 activity are likely distinct between normal development and malignant progression.

## 4 The role of METTL5 in cancer progression

**FIGURE 2:**
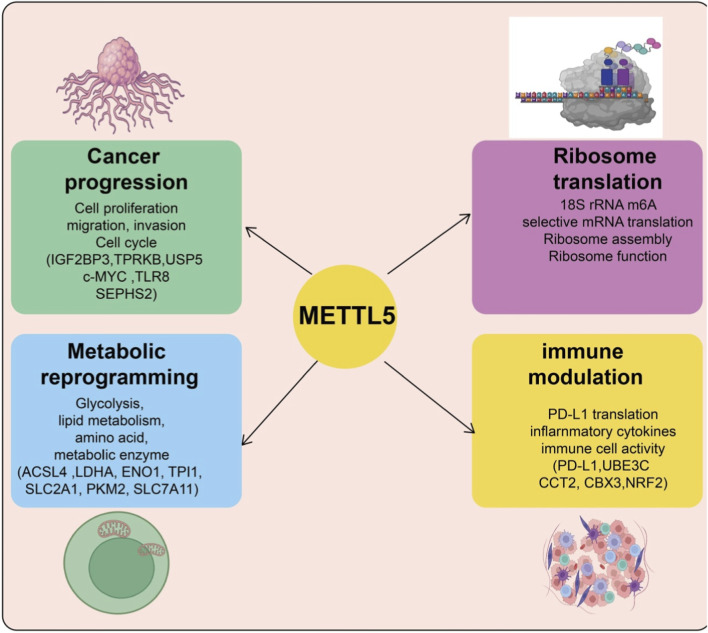
Multifaceted roles of METTL5 in cancer progression. METTL5 promotes various aspects of tumor progression through the regulation of translation or expression of its target genes. In cancer cells, METTL5 enhances cell proliferation, migration, and invasion (IGF2BP3, TPRKB, USP5, c-MYC, TLR8, SEPHS2), drives metabolic reprogramming by modulating glycolysis and lipid metabolism (ACSL4, LDHA, ENO1, TPI1, SLC2A1, PKM2, SLC7A11), regulates ribosome translation and assembly via 18S rRNA m^6^A modification and selective mRNA translation, and modulates immune responses through factors such as PD-L1, UBE3C, CCT2, CBX3, and NRF2. Collectively, these coordinated mechanisms facilitate tumor growth, metabolic adaptation, immune evasion, and therapeutic resistance.

In recent years, accumulating evidence has revealed that METTL5, as an 18S rRNA m^6^A methyltransferase, plays a crucial oncogenic role in the initiation and progression of various cancers. METTL5 is frequently upregulated across multiple malignancies and promotes tumor development through various mechanisms. Its core functions include translational regulation, metabolic reprogramming, ferroptosis inhibition, and chemoresistance. By catalyzing 18S rRNA m^6^A modification, METTL5 enhances the translation efficiency of specific mRNAs, thereby promoting proliferation, migration, and invasion (Dai et al., 2023; Rong et al., 2020; Zhang et al., 2024; Gong et al., 2024). It also stabilizes key proteins such as c-Myc, IGF2BP3, and TPRKB, regulating glycolysis, lipid metabolism, and membrane lipid metabolism to achieve metabolic reprogramming (Xing et al., 2020; Gong et al., 2024; Luo et al., 2024). In addition, METTL5 suppresses ferroptosis by stabilizing antioxidant factors including NRF2, SLC7A11, and UBE3C, thereby facilitating tumor immune evasion (Li et al., 2024; Dong et al., 2025; Chen et al., 2025). Under chemotherapeutic stress, METTL5 increases tumor cell resistance through selective translation of 5′TOP mRNAs or activation of the HSF4b–HSP90B1–mutp53 axis (Chen et al., 2023). The diverse oncogenic roles and underlying mechanisms of METTL5 across different cancer types are summarized in Table 1. These mechanisms are highly conserved across multiple cancer types, including ICC, GC, HCC, NSCLC, CRC, MM, PC, OSCC, LUSC, and NPC, highlighting the potential of METTL5 as a diagnostic biomarker and therapeutic target. Figure 2 summarizes the multifaceted roles of METTL5 in cancer progression, illustrating how it promotes tumor growth, metabolic reprogramming, ribosome function, and immune modulation through regulation of its target genes.

**TABLE 1:** Roles and mechanisms of METTL5 in different cancers.

Cancer type	Expression	Mechanisms	Function	Clinical significance
ICC	Upregulated	18S rRNA m^6^A → translation of TGF-β pathway G-quadruplex mRNAs	Promotes proliferation, migration, invasion	High expression correlates with poor prognosis
GC	Upregulated	rRNA m^6^A regulates protein translation; affects sphingolipid metabolism	Promotes proliferation, migration, invasion; chemoresistance	Associated with advanced stage, distant lymph node metastasis, high pathological grade
NSCLC	Upregulated	Regulates IGF2BP3 expression	Promotes proliferation	High expression correlates with poor prognosis
HCC	Upregulated	Stabilizes c-Myc and 18S rRNA m^6^A → regulates glycolysis and lipid metabolism	Promotes metabolic reprogramming and proliferation	Potential therapeutic target
CRC	Upregulated	Upregulates TLR8	Promotes proliferation and invasion	High expression correlates with poor prognosis
MM	Upregulated	Impaired SEPHS2 translation → increased ROS → apoptosis	Affects selenoprotein synthesis and redox homeostasis	METTL5 inhibitor (SAC) can suppress progression
PAAD	Upregulated	Cooperates with TRMT112 to enhance c-Myc translation	Promotes proliferation, migration, tumorigenesis	METTL5/c-Myc axis as therapeutic target
OSCC	Upregulated	Activates c-Myc pathway	Promotes proliferation, migration, invasion	High expression correlates with poor prognosis
LUSC	Upregulated	m^6^A-dependent upregulation of DEPDC1 translation	Promotes tumorigenesis	Independent risk factor
HNSC	Upregulated	18S rRNA m^6^A1832 → RPL24 → selective translation of 5′TOP mRNAs; HSF4b–HSP90B1–mutp53 axis	Confers chemoresistance	Potential therapeutic target

## 5 The role and mechanisms of METTL5 in tumor immunity

### 5.1 Effects on immune cell function

Emerging evidence indicates that METTL5 plays an important role in shaping immune cell function and immune-related diseases (Turkalj and Vissers, 2022). In airway allergy (AA) mouse models, METTL5 expression was significantly upregulated in M2 macrophages and positively correlated with Th2 polarization. Under AA conditions, the immunosuppressive capacity of M2 macrophages was impaired; however, deletion of Mettl5 in macrophages restored their immunoregulatory function and alleviated allergic responses (Zheng et al., 2025). Mechanistically, Mettl5 enhanced hypermethylation at the Il10 promoter, thereby reducing IL-10 expression (Zheng et al., 2025). Moreover, Mettl5 recruited USP21 to deubiquitinate GATA3 in response to house dust mite extract, leading to elevated IL-4 expression and further strengthening Th2 polarization. Thus, Mettl5 acts as a positive regulator of airway allergy by weakening M2 immunosuppression and promoting Th2 responses, suggesting that its inhibition may represent a therapeutic strategy for allergic diseases (Zheng et al., 2025). In contrast, in CD4^+^ T cells of AA mice, Mettl5 expression was significantly reduced and negatively correlated with AA responses (Miao et al., 2025). Excessive ubiquitination of Mettl5 protein, mediated by the E3 ligase TRIM28, promoted its degradation, which in turn caused hypomethylation and hyperactivation of the Gata3 promoter, triggering spontaneous Th2 polarization (Miao et al., 2025). TRIM28 inhibition restored Mettl5 activity, normalized Gata3 transcription, and ameliorated AA symptoms. These findings highlight the complex context-dependent roles of Mettl5 in regulating immune polarization. In the cancer setting, pan-cancer database analyses have linked METTL5 expression to immune regulation. In renal cell carcinoma (RCC), METTL5 was significantly overexpressed in both clear cell RCC (KIRC) and papillary RCC (KIRP), correlating with tumor stage, grade, and poor prognosis. Co-expression and enrichment analyses revealed associations with Th1/Th2/Th17 differentiation and PI3K-related immune pathways (Wei et al., 2022). Importantly, METTL5 expression was strongly correlated with immune scores, stromal scores, and infiltration levels of 25 immune cell subsets, indicating that METTL5 may promote RCC progression by remodeling the tumor immune microenvironment (TIME). Similar associations were observed in hepatocellular carcinoma (HCC). METTL5 expression was markedly upregulated at the genomic, transcript, and protein levels, accompanied by increased promoter methylation. High METTL5 expression correlated with unfavorable prognosis. Functional enrichment indicated its involvement in ribosome biogenesis, oxidative phosphorylation, mismatch repair, and spliceosome pathways. Notably, bioinformatics analyses (TIMER and TISIDB) revealed positive correlations between METTL5 expression and immune infiltration of B cells, CD8^+^ and CD4^+^ T cells, macrophages, neutrophils, and dendritic cells, as well as significant associations with immune regulatory molecules, chemokines, and their receptors (Wang and Peng, 2023). Collectively, these findings suggest that METTL5 may act as a key modulator of the tumor immune landscape.

Recent studies have identified METTL5 as a key driver of immune resistance in ovarian cancer (Hou et al., 2025). Loss of METTL5 reduces 18S rRNA m^6^A levels, suppresses ATF4 translation and downregulates SLC7A11/SLC3A2, thereby enhancing ferroptosis and T cell-mediated antitumor immunity (Hou et al., 2025). Notably, either ATF4 overexpression or ferroptosis inhibition can reverse the immune-sensitive phenotype induced by METTL5 deficiency, suggesting that the METTL5-ATF4-ferroptosis axis may represent a promising therapeutic target to improve the efficacy of immune checkpoint blockade.

### 5.2 Association with immune checkpoint inhibitor (ICI) response

Recent studies have uncovered an important role of METTL5 in modulating ferroptosis and T cell–mediated antitumor immunity. In gastric cancer (GC), high METTL5 expression was associated with poor prognosis. Functionally, METTL5 suppressed ferroptosis and dampened the cytotoxic activity of peripheral blood mononuclear cells (PBMCs). Mechanistically, METTL5 maintained NRF2 mRNA stability in an m^6^A-dependent manner via the reader protein IGF2BP1, forming a METTL5/m^6^A/NRF2 axis that inhibited Fe^2+^ accumulation, ferroptosis, and T cell–mediated tumor killing (Li et al., 2024). Interestingly, treatment with the ferroptosis inhibitor Ferrostatin-1 reduced PBMC cytotoxicity, suggesting that ferroptosis enhancement could potentiate immunotherapy efficacy. In hepatocellular carcinoma, METTL5 was shown to promote immune evasion through immune checkpoint regulation. Specifically, METTL5 activated the Myc pathway, thereby upregulating PD-L1, c-Myc, CCT2, and CBX3 expression. Chromatin immunoprecipitation and luciferase assays confirmed that PD-L1 expression was driven by Myc promoter activation (Xu et al., 2023). Rescue experiments further demonstrated that Myc overexpression counteracted the suppressive effects of METTL5 knockdown on PD-L1 and tumor cell malignancy. These findings highlight a METTL5–Myc–PD-L1 axis that facilitates HCC immune escape and suggest METTL5 as a potential target for immune checkpoint blockade therapy (Xu et al., 2023). Additionally, natural compounds may target METTL5 to overcome immune resistance. Scutellarin, a flavonoid with anticancer properties, was found to inhibit proliferation, migration, and organoid growth in ovarian cancer models. Mechanistically, Scutellarin downregulated METTL5 expression, and rescue experiments confirmed that its anti-migratory effects were METTL5-dependent (Ding et al., 2025). Thus, targeting METTL5 with small molecules may represent a novel therapeutic avenue in immuno-oncology.

In summary, accumulating evidence highlights METTL5 as a critical regulator of tumor immunity through multiple mechanisms. First, METTL5 modulates immune cell differentiation and function, influencing macrophage polarization, Th2 responses, and CD4^+^ T cell activity in inflammatory and allergic conditions (Wang and Peng, 2023). In cancers such as RCC and HCC, its aberrant expression correlates with immune infiltration, stromal remodeling, and immunoregulatory signaling, underscoring its role in shaping the tumor immune microenvironment. Second, METTL5 mediates immune evasion by suppressing ferroptosis and attenuating T cell–mediated antitumor immunity, largely through the m^6^A-dependent stabilization of NRF2 mRNA. Moreover, the METTL5–Myc–PD-L1 axis provides a direct link between RNA modification and immune checkpoint regulation, highlighting its relevance in resistance to immune checkpoint blockade therapies. Finally, pharmacological inhibition of METTL5, exemplified by natural compounds such as Scutellarin, demonstrates promising antitumor and immunomodulatory effects, suggesting that targeting METTL5 may enhance the efficacy of existing immunotherapies. Collectively, these findings establish METTL5 as a pivotal modulator of tumor immunity and a potential therapeutic target for overcoming immune resistance.

## 6 Discussion and perspectives

In recent years, METTL5 has emerged as a pivotal methyltransferase catalyzing m6A modification at 18S rRNA, and its biological functions are gradually being elucidated (Lei et al., 2023). Although extensive studies have demonstrated the critical roles of METTL5 in maintaining ribosome structural integrity, regulating selective translation, and promoting disease progression, current research remains fragmented and often redundant (Wang et al., 2025). For instance, in multiple disease models, including cancer, metabolic disorders, and neurological diseases, METTL5 is frequently attributed to similar mechanisms such as modulating translation efficiency, influencing stress responses, and promoting cell proliferation (Dai et al., 2023; Xia et al., 2023; Zhang et al., 2024). However, these mechanisms likely stem from a highly overlapping and broadly conserved molecular framework. Therefore, future studies should aim to integrate data across different disease contexts to systematically map the shared regulatory networks of METTL5, thereby reducing mechanistic redundancy and providing a more coherent understanding of its core functions.

First, the function of METTL5 appears to be highly cell type-specific and context-dependent. In different tumor types, its role can be even opposite: in some cancers, METTL5 acts as an oncogene by enhancing the translation of pro-tumor transcription factors, whereas in other tumors, its loss impairs cell survival, reflecting a role in maintaining basal translational homeostasis (Dai et al., 2023; Huang et al., 2022). Interestingly, METTL5 appears to regulate the same oncogenic target, such as MYC, across multiple cancer types (Xia et al., 2023; Xu et al., 2023; Huang et al., 2022). This suggests that METTL5 may exert a conserved pro-tumorigenic role by modulating the translation or expression of key drivers like MYC, regardless of tissue context. The consistent regulation of MYC by METTL5 highlights its potential as a broad-spectrum therapeutic target, as inhibiting METTL5 could simultaneously disrupt oncogenic programs in diverse malignancies. Furthermore, these observations raise the possibility that METTL5-mediated translational control represents a common mechanistic node linking tumor growth, metabolism, and proliferation across cancers. This variability may arise from tumor heterogeneity, including differences in metabolic microenvironments, RNA-binding protein repertoires, signaling pathway activities, and ribosome composition. Moreover, under distinct physiological or pathological stimuli, the downstream mRNA targets of METTL5 may shift substantially, mediating diverse functional outcomes (Zhang et al., 2025). Therefore, future research should systematically compare METTL5 target mRNA profiles, sequence-structure dependencies, and cell-state specificity across a wide spectrum of tissues and disease models to establish a unified, cross-disease regulatory framework.

Second, METTL5 functions likely extend beyond classical translational control. During ribosome biogenesis and nuclear RNA metabolism, METTL5 may participate in broader RNA regulatory and chromatin dynamics. Emerging evidence suggests that METTLs may possess the capacity for liquid–liquid phase separation (LLPS), potentially contributing to nucleolar integrity, ribosome assembly center clustering, and RNA–protein condensate formation. If METTL5 engages in LLPS-mediated ribosome biogenesis or selective mRNA translation, it would introduce a novel dimension to its functional repertoire, warranting further exploration using super-resolution microscopy, *in vitro* reconstitution systems, and LLPS perturbation experiments. Furthermore, METTLs may interact with three-dimensional genome organization. Previous studies indicate close connections among ribosome biogenesis, chromatin folding, and nucleolar-associated chromatin domains. By regulating ribosome biogenesis rates, nucleolar structural stability, and the modification of chromatin-associated RNAs, METTL5 may indirectly influence higher-order chromatin architecture. For example, its modification of specific noncoding RNAs or chromatin-anchored RNAs could affect chromatin accessibility, spatial distribution of transcription factories, or enhancer–promoter interactions. Investigating whether METTL5 directly participates in chromatin RNA regulation and modulates 3D genome structure represents a critical future research direction.

Targeting METTL5 inhibition may hold substantial therapeutic potential in cancer progression. As METTL5 promotes immune resistance through sustaining ATF4 translation and suppressing ferroptosis, its inhibition could shift tumors toward a more immunogenic state, thereby enhancing responsiveness to immune checkpoint blockade (Hou et al., 2025). In addition to immunomodulation, METTL5 blockade may also sensitize cancer cells to ferroptosis-inducing agents and metabolic stress, suggesting a synergistic strategy when combined with existing therapies. Given the elevated METTL5 expression in clinically “cold” tumors and poor responders, selective inhibition of METTL5 may provide a rational approach to overcome tumor immune evasion and broaden the efficacy of immunotherapy across otherwise refractory cancers.

Emerging evidence indicates that METTL5 exerts biological functions beyond its canonical methyltransferase activity. Several studies have suggested that METTL5 may serve as a structural component of ribosomal complexes, contributing to ribosome stability or selective mRNA translation in a manner that does not strictly rely on its catalytic function. In addition, non-enzymatic roles of METTL5 have been implicated in stress response pathways, including ATF4-mediated translational control, raising the possibility that protein–protein interactions may underlie these effects. However, whether these non-catalytic activities are preserved across cancer progression and tumor immunity remains largely unexplored. Given the importance of METTL5 in shaping immune evasion, elucidating the relative contribution of enzymatic versus structural functions will be essential to define its mechanisms in oncogenesis and to guide the development of targeted therapies.

At the disease level, METTL5’s role in the tumor immune microenvironment remains largely unexplored. Immune cell types such as dendritic cells, macrophages, and T cells are highly sensitive to ribosome function and translational regulation, yet METTL5’s activity in these cells is virtually uncharacterized (Wang and Peng, 2023). METTL5 may influence the translation of key immune modulators, including cytokines, co-stimulatory molecules, or immunosuppressive factors, thereby affecting immune infiltration, anti-tumor immunity, and immunotherapy responsiveness. Comprehensive dissection of METTL5 function in immune cells may reveal novel combinatorial targets for cancer immunotherapy. In terms of clinical translation, targeting METTL5 with small-molecule inhibitors presents a promising therapeutic avenue. Given its relatively simple structure and well-defined catalytic site, METTL5 is amenable to drug development. The integration of artificial intelligence tools and structure-prediction algorithms such as AlphaFold offers new opportunities to accelerate rational design, virtual screening, and dynamic modeling for highly selective inhibitors or degraders (Abramson et al., 2024). Considering the context-dependent activity of METTL5, future drug development should prioritize stratification of METTL5-dependent tumor subtypes, leveraging CRISPR gene dependency data, compound sensitivity profiles, and single-cell transcriptomics to enable precision targeting. Collectively, METTL5 represents a highly complex and multilayered regulatory node, with functions spanning classical rRNA modification and translational control to phase separation, chromatin architecture, immune microenvironment regulation, and tumor heterogeneity. Future studies should adopt interdisciplinary, multi-dimensional approaches to integrate these mechanisms, thereby providing a comprehensive understanding of METTL5 biology and enabling its translation into disease diagnosis, risk assessment, and targeted therapeutic strategies.
